# A Cost and Cost-Benefit Analysis of the Stand More AT Work (SMArT Work) Intervention

**DOI:** 10.3390/ijerph17041214

**Published:** 2020-02-13

**Authors:** Fehmidah Munir, Paul Miller, Stuart J.H. Biddle, Melanie J. Davies, David W. Dunstan, Dale W. Esliger, Laura J. Gray, Sophie E. O’Connell, Ghazala Waheed, Thomas Yates, Charlotte L. Edwardson

**Affiliations:** 1 School of Sport, Exercise and Health Sciences, Loughborough University, Loughborough LE11 3TU, UK; D.Esliger@lboro.ac.uk; 2 Miller Economics Ltd., Biohub Alderley Park, Alderley Edge SK10 4TG, UK; drpsjmiller@gmail.com; 3 Institute for Resilient Regions, University of Southern Queensland, Education City, Springfield Central, QLD 4300, Australia; Stuart.Biddle@usq.edu.au; 4 Diabetes Research Centre, University of Leicester, Leicester General Hospital, Leicester LE5 4PW, UK; Melanie.davies@uhl-tr.nhs.uk (M.J.D.); Gw136@leicester.ac.uk (G.W.); ty20@le.ac.uk (T.Y.); 5 NIHR Leicester Biomedical Research Centre, Leicester General Hospital, Leicester LE5 4PW, UK; 6 Leicester Diabetes Centre, University Hospitals of Leicester, Leicester General Hospital, Leicester LE5 4PW, UK; Sophie.OConnell@uhl-tr.nhs.uk; 7 Baker Heart and Diabetes Institute, Melbourne, VIC, Australia; David.Dunstan@bakeridi.edu.au; 8 Mary MacKillop Institute for Health Research, The Australian Catholic University, Melbourne, VIC 3000, Australia; 9 Department of Health Sciences, University of Leicester, Leicester LE1 7RH, UK; lg48@leicester.ac.uk

**Keywords:** cost-benefit analysis, sitting, standing, sit-stand, presenteeism, sick leave, sickness absence, workplace health promotion

## Abstract

This study conducted a cost and cost-benefit analysis of the Stand More AT (SMArT) Work workplace intervention, designed to reduce sitting time. The study was a cluster two-armed randomised controlled trial involving 37 office clusters (146 desk-based workers) in a National Health Service Trust. The intervention group received a height-adjustable workstation with supporting behaviour change strategies. The control group continued with usual practice. Self-report absenteeism, presenteeism and work productivity were assessed at baseline, 3, 6 and 12 months; and organisational sickness absence records 12 months prior to, and 12 months of the intervention. Mean per employee costs associated with SMArT Work were calculated. Absenteeism, presenteeism and work productivity were estimated, and employer-recorded absence data and employee wage-banding were used to provide a human-capital-based estimate of costs to the organisation. The return-on-investment (ROI) and incremental cost-efficacy ratios (ICER) were calculated. Intervention cost was £692.40 per employee. Cost-benefit estimates show a net saving of £1770.32 (95%CI £-354.40, £3895.04) per employee as a result of productivity increase. There were no significant differences in absence data compared to the control group. SMArT Work provides supporting evidence for policy-makers and employers on the cost benefits of reducing sitting time at work.

## 1. Introduction

High levels of sedentary behaviour (sitting time) have been identified as an important modifiable behavioural risk factor for multiple chronic diseases [1,2,3,4], poor mental health [5,6] and premature mortality [4]. In office workers, workplace sitting accounts for the largest proportion of daily sitting time [7]; with 70%-85% of time spent sitting at work, and over a third of that total sitting time undertaken in bouts of over 30 min at a time—prolonged sitting [8,9]. With problems such as neck and shoulder pain, high presenteeism and low work engagement associated with sitting time at work [10,11,12], the office workplace is a priority setting to reduce total occupational sitting time [13]. Whilst workplace interventions have shown that significant short and medium-term reductions in workplace sitting time can be achieved [8,14,15,16,17], few have reported on the cost-benefits of their interventions [18], and this lack of evidence may act as a barrier for organisations in adopting and promoting strategies to reduce sitting time at work. Evaluating the cost-benefits of reducing occupational sitting time are important due to the estimated costs of £30bn ($39bn; €34bn) for sickness presenteeism (i.e., working despite being unwell to work) [19], and sickness absenteeism in the United Kingdom, with presenteeism costing twice as much as absenteeism [20].

The Stand More AT Work (SMArT Work) intervention is a multicomponent intervention designed to reduce occupational sitting time. It was tested within a cluster randomised controlled trial over 12 months in a sample of office workers working within the English National Health Service. The intervention successfully reduced sitting time over the short, medium and longer term, and led to positive changes in work related and psychological health [21].

This study reports the cost-benefit analysis of the SMArT Work intervention by providing an assessment of the costs associated with implementing the intervention over a 12- month period. The cost-analysis was conducted from the perspective of the employer—who generally pays the costs of implementing workplace interventions—and the intervention costs were measured against a control group. The aim of the study was to provide accurate cost data and the cost-benefit of the intervention. This will support both employers and public health policy makers in drawing evidence-based decisions concerning the economic feasibility and scalability of reducing workplace sitting time.

## 2. Materials and Methods 

### 2.1. Study Design

A detailed description of the intervention development, randomised controlled trial design and main results, have been published previously [21,22]. Briefly, SMArT Work was a two-arm cluster randomised controlled trial, consisting of an intervention group and a control group. Randomization conducted at office level at a ratio of 1:1. Participants in the intervention group received a height-adjustable workstation (a choice of workstation—a full, electrical desk or VARIDESK desk platform) as well as supporting behaviour change strategies including education, behavioural feedback, self-monitoring and prompt tool (DARMA cushion), quarterly coaching sessions and information leaflets and motivational posters aimed at reducing sitting time in staff who were predominantly desk-based. The control group continued in their usual working environment. 

### 2.2. Ethical Considerations

All subjects gave their informed consent for inclusion before they participated in the study. The study was conducted in accordance with the Declaration of Helsinki. This study was approved by Loughborough University, and Research and Innovation approval was obtained from the University Hospitals of Leicester NHS Trust (EDGE ID 34571).

### 2.3. Analyses Approach

A simple cost-benefit analysis was constructed by synthesizing: a) cost analyses—the mean per employee costs associated with implementing the SMArT Work intervention; and b) organizational effect measures—the change in absenteeism, presenteeism and overall work productivity gain/loss expressed in terms of time and estimated costs to the organization. Where both costs and organizational effect measures were estimated in money terms, the net effect in terms of cost-benefit was calculated (i.e., to what extent are benefits/effects expected to outweigh the costs, and the estimated return-on-investment (ROI)).

### 2.4. Intervention Costs

The total and per employee cost for providing this intervention package for the 12-month study period was calculated. The costs of the intervention were estimated based on material costs and research facilitator costs to implement the intervention. Material costs included the expense of the height adjustable workstation, the DARMA cushion (self-monitoring and prompt tool) and printing costs of supporting documents, i.e., action plan and goal setting diary, feedback sheets on behaviour, posters and education material. Facilitator costs, estimated using the facilitator’s hourly wage (£18.20), included removal of the existing desk for those choosing the electric height adjustable desk, installation of the height adjustable workstation, including demonstration on how to use it, production of behaviour feedback, coaching sessions and delivery of an educational seminar. Indirect work loss costs associated with participant engagement with the intervention (e.g., attendance at seminar) was estimated using the mean hourly wage rate for the intervention cohort (£16.01) based on the mid-point of each wage band.

### 2.5. Measures of Effects

Absenteeism (work time missed), presenteeism (impairment at work/reduced on-the-job effectiveness), work productivity (overall change in productivity/absenteeism plus presenteeism) and activity impairment were estimated via self-report using the Work Productivity and Activity Impairment Questionnaire: General Health V2.0 (WPAI:GH) [23]. Absolute estimates of absenteeism, presenteeism and work productivity from the WPAI measure were multiplied by individual employee wage-banding information to provide a human-capital-based estimate of costs to the employing organization. WPAI-based time and cost data for the intervention and control groups were compared at the four time points (baseline, 3 months, 6 months and 12 months). Analyses were based on the mean of these time points. Productivity and sickness absence were analysed using Generalized Estimating Equation (GEE) model with an exchangeable correlation structure and accounting for clustering. The model included a binary indicator for randomization group and adjusted for cluster size (<= 4 participants or > 4 participants). We also adjusted for sex and ethnicity as these differed between the intervention and control group at baseline, with more males (27.3 vs. 13.0%) and South Asians (20.8 vs. 13.0%) represented in the intervention group Analyses were conducted using Stata version 14. 

Objectively measured absenteeism data were also collected through employer-recorded absence for the intervention and control participants who consented and were compared for two time periods: 12 months prior to the study initiation (period 1) and for the 12 months of the intervention and follow-up (period 2). The change from time period 1 to 2 was compared across the two groups. Absolute measures of absenteeism based on employer-recorded data were multiplied by individual employee wage-banding information to provide a human-capital-based estimate of costs to the employing organization. 

### 2.6. Cost Benefit Analysis

All the economic calculations presented are based on the full cohort data for control and intervention. Two separate economic analyses were conducted based on the two distinct sources of data to estimate measures of organizational effects (self-report vs. archival data). In both analyses, measures of effects (change in work loss time) were monetized using the human-capital-based method applying the employee’s wage rate to value their productive time at work.

The net impact in terms of cost-benefit was also calculated, only where benefits are found to outweigh costs this net impact will be a positive number. The ratio of costs to monetized benefits were used to calculate the return-on-investment (ROI). Net impact cost-benefit and ROI were calculated for the whole intervention group compared to controls defined by key parameters including observed degree of exposure [21].

Finally, incremental cost-efficacy ratios (ICER) were calculated as the difference in costs of the intervention and control group divided by the difference in their effect. The ICER was expressed as the cost per unit (minutes per workday) reduction in workplace sitting time at 12 months. Both complete case and Intention to treat data imputed using multiple imputation [21], were used for ICER calculations. Means of normal distributions and standard distributions were used (the latter expressed as the mean of non-zero) for both the intervention and control group.

## 3. Results

### 3.1. Intervention Costs

Table 1 shows the breakdown of the intervention costs. Overall intervention costs were calculated at £595 per participating employee, with ~90% of costs attributed to equipment (workstation and cushion). Table 2 shows the indirect work loss costs associated with participant work time spent engaging with the intervention, estimated at £97.40 per participant over the 12 months. The aggregate costs of the intervention (direct + indirect) over the 12-month period was calculated as £595 + £97.40 = £692.40 per participant.

### 3.2. Measures of Effects

Table 3 shows self-reported absence and productivity. Results show employee productivity improved in the intervention group (mean 1.75hrs better per week) and worsened in the control group (mean 0.44hrs worse) from baseline to 12 months. Significant differences between the groups were found with respect to the cost of self-reported productivity: adjusted difference mean £47.36 (95%CI £6.50, £88.22) savings for past 7 days. There were no significant differences in self-reported absenteeism due to health reasons between the intervention and control groups nor when archival absence data were used (data not shown).

### 3.3. Cost Benefit

In terms of cost-benefit analyses, the delta costs and effects observed between the intervention and control groups are as follows:

1. ∆cost = + £595 (intervention costs) + £97.40 (indirect work loss costs) = + £692.40
2. ∆effect (1) = WPAI productivity gain £47.36 per week x 52 weeks = £2462.72
3. (95%CI∆ £6.50, £88.22 per week x 52 weeks = £338; £4587.44)
4. ∆effect (2) = Archival sickness absence = no difference
5. ∆effect (3) = WPAI sickness absence = no difference

Therefore, the SMArT Work intervention was estimated to provide a net benefit in productivity per intervention group employee over 52 weeks of £1770.32 (£2462.72 - £692.40) (95%CI∆ £-354.40, £3895.04). This equates to a return on investment of 256% ((£2462.72-692.40) / £692.40 *100). The productivity gains associated with SMArT Work (Table 3 - £47.36 savings for past 7 days), equates to mean wage rates of £29.97. Where productivity gains associated with the intervention are circa 2 hours per week and hourly wage rates vary between £10 and £30, savings will be between £20 and £60 per week per employee.

In terms of cost per unit (minutes per workday) reduction in workplace sitting time at 12 months, the ICER for the SMArT Work intervention is £8.31 (using complete case data), £8.48 (using intention to treat data) and £16.77 (standardised to 8h workday). 

## 4. Discussion

This analysis reports the costs of a multi-component intervention to reduce sitting time in desk-based workers and the cost-benefit from an employer perspective. When considering all intervention components, regardless of whether the participants engaged with them, the intervention was estimated to cost £692.40 per person, with approximately 90% of these costs attributed to the height-adjustable workstation and Darma cushion. The intervention is estimated to provide a net benefit (saving) per employee, calculated over the course of the 12 month randomised controlled trial, of £1770.32 (mean of all follow up time points) as a result of reduction in productivity loss due to health problems. The return on investment is 256%. In other words, £2.56 would be returned for every £1 spent on the intervention.

The results of the randomised controlled trial [21], showed no difference in the effectiveness of the intervention between the two different types of height-adjustable workstations used. However, there is a substantial price difference between these workstations in addition to installation and removal costs, resulting in the electric workstation being approximately £150 more expensive than the platform workstation. Furthermore, our process evaluation found the majority of participants did not use the self-monitoring device (Darma cushion) and nearly 25% of the total intervention costs were attributed to this device. Some participants chose not to self-monitor or use formal prompts, whereas others sought their own free methods such as electronic timers and computer software for tracking their sitting time and providing reminders to break up their sitting. If the intervention was costed based on the platform workstation and the use of free self-monitoring and prompt methods, the cost of the intervention would reduce from £692.40 to £430.87 per person (intervention and work loss costs included) and the return on investment would increase to 472% (£4.72 returned for each £1 spent). Furthermore, it can be reasonably expected that as height-adjustable workstations become more common, prices will continue to fall, which will increase the return on investment. 

The primary outcome of the study was change in occupational sitting time at 12-month follow up [21]. A significant difference between groups (in favour of the intervention group) was found in occupational sitting time at 12 months (− 83.28 min/workday, 95% confidence interval − 116.57 to − 49.98, *p* = 0.001). In terms of cost per unit (minutes per workday) reduction in workplace sitting time at 12 months, the ICER for the SMArT Work intervention is £8.31 (complete case), £8.48 (ITT population) and £16.77 (standardised to 8h workday). There are a lack of studies reporting intervention costs and benefits, however, one recently published study reported the economic evaluation of the Stand Up Victoria sitting reduction intervention in Australia (Gao et al 2018). Intervention costs were reported at $431AUD (~£235) per participant and the ICER was AU$9.94 (~£5.49) cost per minute reduction in workplace sitting time, which is less than the SMArT Work intervention costs. There are some explanations for this. Although, both interventions were multi-component interventions including a height-adjustable desk per participant, different types of desks were used across these studies, which varied in price. Furthermore, other intervention components, although similar (e.g., education) were implemented differently. The SMArT Work intervention also included expensive self-monitoring and prompt equipment. These factors will have led to a difference in cost. Moreover, in the Stand Up Victoria study, the cost of the height-adjustable workstations was annuitized over 5 years, this was not done in the current analysis.

This cost analysis was conducted from the perspective of the employer, hence the focus on productivity gains, presenteeism and absenteeism. In a recent paper, Gao and colleagues [24] translated the outcome observed in Stand Up Victoria into costs per life year (LY) gained and costs per health-adjusted life years (HALY) gained and reported higher LY and HALY gains and lower long-term health costs, suggesting that the intervention was cost-effective over the lifetime of the cohort. Subsequently, Gao and colleagues [18], modelled an economic evaluation to simulate the long-term health benefits of workplace interventions targeting a reduction in sitting time in preventing cardiovascular disease (CVD). They showed a resultant ICER of $43,825 per QALY gained, making the intervention effective for the primary prevention of CVD. Despite this recent progress on the cost-benefit, return on investment and cost-effectiveness of workplace sitting reduction interventions, the data are still limited and more evidence is needed to continue to build a stronger business case for organisations to adopt a focus on sitting less in the workplace.

The strengths of this study include the randomised controlled trial, with short and medium term follow up time points, and the inclusion of organization absenteeism records. Presenteeism and work productivity however were assessed via self-report which may introduce bias with employees either over-estimating or under-estimating their productivity. Furthermore, productivity gains were calculated from a questionnaire assessing the past 7 days, completed four times across the 12-month period. These gains were then estimated over 52 weeks and therefore may not extrapolate exactly but the magnitude of net benefit per employee would appear to leave some margin for variance in these estimates. The costs of the intervention are based on the actual facilitator’s and participants’ pay grades, therefore the costs in the intervention may fluctuate depending on who facilitates the implementation and takes part.

## 5. Conclusions

In summary, based on material costs, facilitator costs to implement the intervention and indirect work loss costs the SMArT Work intervention cost £692.40 per person. Despite its high cost, the intervention is estimated to provide a net saving per employee of £1770.32, over an initial 12-month time period, as a result of an increase in productivity. This equates to a £2.56 return on investment for every £1 spent on the intervention. 

## Figures and Tables

**Table 1:** Intervention cost breakdown: Materials and facilitator time.

Item	Unit Cost	Quantity	Total Cost
*Desks ^a^*			
Varidesk 36 Pro Plus	£301.50	38	£11,457
Varidesk 40 Plus	£352.50	7	£2467.50
Varidesk installation	£12.50	45	£562.50
Electric desk	£453.71	30	£13,611.30
Removal of old desk	£18.50	30	£555
Electric desk installation	£7.50	30	£225
**Sub-Total**			**£28,878.30**
*ActivPAL feedback ^b^*			
Report production	£4.55	255	**£1160.25**
*DARMA ^a^*	£169	75	**£12,675**
*Information support ^b^*			
Design and delivery of Seminars	£22.75 ^c^	8	£182
Printing of posters/leaflets/diaries	£6.99	75	£524.25
Researcher demonstration of desk use	£0.91	75	£68
Coaching	£15.16	75	£1,138
**Sub-Total**			**£1912.25**
**TOTAL**			
Per participant			**£44,625.80**
		**75**	**£595**

^a^ RRP; ^b^ based on researcher time £18.20 per hour; ^c^ includes organisation time.

**Table 2:** Participant time spent on intervention and estimated workloss costs.

Intervention Element	Time/cost to participant	Workloss Costing over 12m (@ mean hourly rate £16.02)
Desks set-up and demonstration	Varidesk: 10 min to set up (n = 45) Electric desk: 30 min to remove old desk and set up new desk (n = 30)	Assume 15 mins one-off workloss = £4.00
Seminar	Approx. 45 min of their workday to attend a one off seminar on site	Assume 45mins one-off workloss = £12.02
activPAL feedback reading and reading initial leaflet Self-monitoring sitting via DARMA cushion (per unit) Gola setting diaries	activPAL and leaflet total time per participant is 15 min Utilising DARMA feedback and diaries a total time of 5 min per week per participant	Workloss (50*5mins + 15min = 265mins) = £70.71
Coaching sessions	Brief 10 min with researcher to discuss progress, motivations, goals and plans every 3 months = a total of 40 min over a 12-month period per participant	Assume 40mins one-off workloss = £10.67
**TOTAL**	Intervention cohort (n = 75) Per participant	**£7305** **£97.40**

**Table 3:** Self-reported productivity change and absenteeism due to health problems.

Metrics			Change from Baseline	
			hrs	£’s
**Productivity change**	Intervention	n	65	
		Mean non-zero	2.41	£62.83
		Mean (95% CI)	1.75 (gain) (0.09 to 3.42)	£33.72 (gain) (£60.87 to -£6.58)
	Control	n	52	
		Mean non-zero	-1.07	-£35.21
		Mean (95% CI)	-0.44 (loss) (-2.09 to 1.21)	-£17.68 (loss) (-£50.44 to £15.08)
	Delta (I-C)	Mean (95%CI)	2.19 (-0.16 to 4.55)	£51.41 (£9.68 to £93.13)
**Adjusted difference ^a^**		Mean (95% CI)	1.58 (-0.50 to 3.67)	£47.36 (£6.50 to £88.22)
		***p*-value**	0.137	**0.023**
**Sickness Absence**	Intervention	n	66	
		Mean non-zero	3.65	£46.52
		Mean (95%CI)	0.28 (-0.43 to 0.99)	£5.42 (-£3.76 to £14.61)
	Control	n	52	
		Mean non-zero	7.67	£64.86
		Mean (95%CI)	0.23 (-0.77 to 1.22)	£3.33 (-£8.46 to £15.13)
	Delta (I-C)	Mean (95% CI)	0.05 (-1.12 to 1.23)	£2.09 (-£12.47 to £16.64)
**Adjusted difference ^a^**		Mean (95% CI)	0.07 (-1.27 to 1.41)	£3.82 (-£13.41 to £21.04)
		***p*-value**	0.918	0.664

^a^ Adjusted difference in the productivity and sickness absence between treatment groups (intervention group compared to control group) with 95% confidence interval, *p*-value; adjusted for cluster effect, sex, ethnicity and stratification categories (office size <4 and office size>=4).

## Trial Registration

ISRCTN10967042. Registered on 2 February 2015.

## References

[1] Wilmot E.G., Edwardson C.L., Achana F.A., Davies M.J., Gorely T., Gray L.J., Khunti K., Yates T., Biddle S.J.H. (2012). Sedentary time in adults and the association with diabetes, cardiovascular disease and death: systematic review and meta-analysis. Diabetologia.

[2] De Rezende L.F.M., Rey-López J.P., Matsudo V.K.R., Luiz O.D.C. (2014). Sedentary behavior and health outcomes among older adults: A systematic review. BMC Public Health..

[3] Shen D., Mao W., Liu T., Lin Q., Lu X., Wang Q., Lin F., Ekelund U., Wijndaele K. (2014). Sedentary Behavior and Incident Cancer: A Meta-Analysis of Prospective Studies. PLoS ONE.

[4] Biswas A., Oh P.I., Faulkner G.E., Bajaj R.R., Silver M.A., Mitchell M.S., Alter D.A. (2015). Sedentary time and its association with risk for disease incidence, mortality, and hospitalization in adults: A systematic review and meta-analysis. Ann. Intern. Med..

[5] Teychenne M., A Costigan S., Parker K. (2015). The association between sedentary behaviour and risk of anxiety: A systematic review. BMC Public Health..

[6] Zhai L., Zhang Y., Zhang D. (2015). Sedentary behaviour and the risk of depression: A meta-analysis. Br. J. Sports Med..

[7] Parry S., Straker L. (2013). The contribution of office work to sedentary behaviour associated risk. BMC Public Health..

[8] Healy G.N., Eakin E.G., Lamontagne A.D., Owen N., Winkler E.A., Wiesner G., Gunning L., Neuhaus M., Lawler S., Fjeldsoe B.S. (2013). Reducing sitting time in office workers: Short-term efficacy of a multicomponent intervention. Prev. Med..

[9] Clemes S.A., O’connell S.E., Edwardson C.L. (2014). Office Worker’s Objectively Measured Sedentary Behavior and Physical Activity During and Outside Working Hours. J. Occup. Environ. Med..

[10] Hallman D.M., Gupta N., Mathiassen S.E., Holtermann A. (2015). Association between objectively measured sitting time and neck–shoulder pain among blue-collar workers. Int. Arch. Occup. Environ. Health.

[11] Munir F., Houdmont J., Clemes S., Wilson K., Kerr R., Addley K. (2015). Work engagement and its association with occupational sitting time: Results from the Stormont study. BMC Public Health..

[12] Brown H.E., Ryde G.C., Gilson N.D., Burton N.W., Brown W.J. (2013). Objectively measured sedentary behavior and physical activity in office employees: Relationships with presenteeism. J. Occup. Environ. Med..

[13] Neuhaus M., Healy G.N., Fjeldsoe B.S., Lawler S., Owen N., Dunstan D.W., Lamontagne A.D., Eakin E.G. (2014). Iterative development of Stand Up Australia: A multi-component intervention to reduce workplace sitting. Int. J. Behav. Nutr. Phys. Act..

[14] Healy G.N., Winkler E.A., Eakin E.G., Owen N., Lamontagne A.D., Moodie M., Dunstan D.W. (2017). A cluster RCT to reduce workers’ sitting time: Impact on cardio-metabolic biomarkers. Med. Sci. Sports Exerc..

[15] Karakolis T., Callaghan J.P. (2014). The impact of sit–stand office workstations on worker discomfort and productivity: A review. Appl. Ergon..

[16] Danquah I.H., Kloster S., Holtermann A., Aadahl M., Bauman A., Ersbøll A.K., Tolstrup A.S. (2017). Take a Stand!-a multi-component intervention aimed at reducing sitting time among office workers-a cluster randomized trial. Int. J. Epidemiol..

[17] Dunstan D.W., Wiesner G., Eakin E.G., Neuhaus M., Owen N., Lamontagne A.D., Moodie M., Winkler E.A., Fjeldsoe B.S., Lawler S. (2013). Reducing office workers’ sitting time: Rationale and study design for the Stand Up Victoria cluster randomized trial. BMC Public Health..

[18] Gao L., Nguyen P., Dunstan D., Moodie M. (2019). Are Office-Based Workplace Interventions Designed to Reduce Sitting Time Cost-Effective Primary Prevention Measures for Cardiovascular Disease? A Systematic Review and Modelled Economic Evaluation. Int. J. Environ. Res. Public Health.

[19] Aronsson G., Gustafsson K. (2005). Sickness presenteeism: Prevalence, attendance-pressure factors, and an outline of a model for research. J. Occup. Environ. Med..

[20] ERS Research and Consultancy (2016). Health at Work Economic Evidence Report. https://www.bhf.org.uk/informationsupport/publications/health-at-work/health-at-work---economic-evidence-report.

[21] Edwardson C.L., Yates T., Biddle S.J.H., Davies M.J., Dunstan D.W., Esliger D.W., Gray L.J., Jackson B., E O’Connell S., Waheed G. (2018). Effectiveness of the Stand More AT (SMArT) Work intervention: Cluster randomised controlled trial. BMJ.

[22] O’Connell S.E., Jackson B.R., Edwardson C.L., Yates T., Biddle S.J.H., Davies M.J., Dunstan D., Esliger D., Gray L., Miller P. (2015). Providing NHS staff with height-adjustable workstationsand behaviour change strategies to reduce workplace sitting time: Protocol for the Stand More AT (SMArT) Work cluster randomised controlled trial. BMC Public Health.

[23] Reilly M.C., Zbrozek A.S., Dukes E.M. (1993). The Validity and Reproducibility of a Work Productivity and Activity Impairment Instrument. Pharmacoeconomics.

[24] Gao L., Flego A., Dunstan D.W., Winkler E.A., Healy G.N., Eakin E.G., Willenberg L., Owen N., Lamontagne A.D., Lal A. (2018). Economic evaluation of a randomized controlled trial of an intervention to reduce office workers’ sitting time: The "Stand Up Victoria" trial. Scand. J. Work. Environ. Health..

